# Supplementary material for: Insights into the Photoelectrocatalytic Behavior of gCN-Based Anode Materials Supported on Ni Foams
Source: Nanomaterials (Basel). 2023 Mar 13;13(6):1035. doi: 10.3390/nano13061035 (PMC10057045; doi:10.3390/nano13061035)
Supplement: Supplementary file 1 [file nanomaterials-13-01035-s001.zip › nanomaterials-2257165-supplementary.pdf]

Supporting Materials

Insights into the Photoelectrocatalytic Behavior of gCN-Based Anode Materials Supported on Ni Foams

Serge Benedoue [1,2], Mattia Benedet [1], Alberto Gasparotto [1,3,*], Nicolas Gauquelin [4], Andrey Orekhov [4], Johan Verbeeck [4], Roberta Seraglia [3], Gioele Pagot [5], Gian Andrea Rizzi [1,3,*], Vincenzo Balzano [6], Luca Gavioli [6], Vito Di Noto [5], Davide Barreca [3] and Chiara Maccato [1,3]

[1] Department of Chemical Sciences, Padova University and INSTM, 35131 Padova, Italy; benedoueserge@yahoo.com (S.B.); mattia.benedet@phd.unipd.it (M.B.); chiara.maccato@unipd.it (C.M.); alberto.gasparotto@unipd.it (A.G.); gianandrea.rizzi@unipd.it (G.A.R.)
[2] Laboratory of Applied Physical and Analytical Chemistry, Department of Inorganic Chemistry, Faculty of Science, University of Yaounde1, Yaoundé P.O. Box 812, Cameroon
[3] CNR-ICMATE and INSTM, Department of Chemical Sciences, Padova University, 35131 Padova, Italy; roberta.seraglia@cnr.it (R.S.); davide.barreca@unipd.it (D.B.)
[4] EMAT and NANOlab Center of Excellence, University of Antwerp, 2020 Antwerpen, Belgium; nicolas.gauquelin@uantwerpen.be (N.G.); andrey.orekhov@uantwerpen.be (A.O.); jo.verbeeck@uantwerpen.be (J.V.)
[5] Section of Chemistry for the Technology (ChemTech), Department of Industrial Engineering, University of Padova and INSTM, 35131 Padova, Italy; gioele.pagot@unipd.it (G.P.); vito.dinoto@unipd.it (V.D.N.)
[6] Interdisciplinary Laboratories for Advanced Materials Physics (i-LAMP), Dipartimento di Matematica e Fisica, Università Cattolica del Sacro Cuore, 25133 Brescia, Italy; vincenzo.balzano@unicatt.it (V.B.); luca.gavioli@unicatt.it (L.G.).

* Correspondence: alberto.gasparotto@unipd.it, gianandrea.rizzi@unipd.it; Tel.: +39-0498275192 (A.G.); +39-0498275722 (G.A.R.).

S1. Experimental details

S1.1. Synthesis

gCN^{Ar} powders were synthesized by thermal condensation of melamine (99%, Sigma-Aldrich) in an Ar atmosphere [1]. Briefly, melamine powders were introduced in a closed crucible, placed in a tubular oven and then heated with a constant rate of 3°C/min at 100°C (30 min), 400°C (2.5 h), and finally 550°C (4 h), followed by slow cooling at room temperature. The preparation of gCN^{air} powders was carried out in a similar way, but operating in air in a muffle furnace. In this case, melamine powders (typically 3 g) were transferred into a closed crucible and heated at a rate of 3°C/min at 100°C (30 min), 400°C (2 h), and then 550°C (4 h), followed by cooling at room temperature.

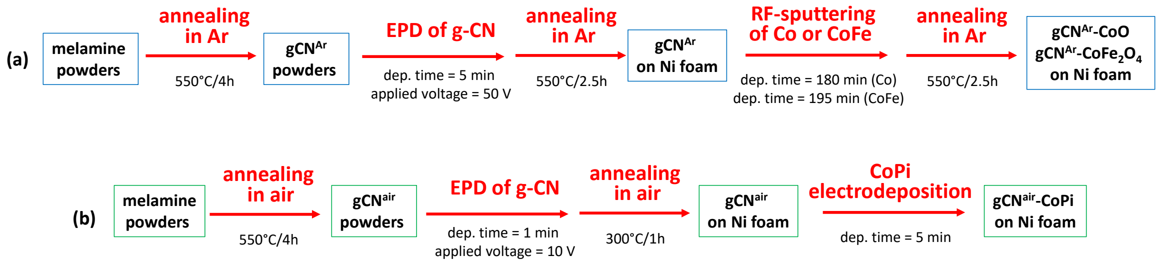

Figure S1. Synthetic protocols used for the preparation of: (a) gCN^{Ar} and (b) gCN^{air} powders, and the corresponding Ni foam-supported samples. The most relevant parameters are also reported.

S1.2. Characterization

High-resolution electron energy loss spectroscopy (EELS) data were acquired on a state-of-the-art double-corrected and monochromated Thermo Fisher Scientific Titan 80-300 microscope operated at 120 kV to limit beam damage while keeping a sub-nm spatial resolution and an energy resolution of 120 meV, a convergence angle of 19 mrad and a collection angle of 90 mrad. EELS spectra were acquired on a Direct detection Gatan K2 camera mounted on a GIF Quantum spectrometer. Dual EELS was used to get absolute energy of the Co *L*, O *K* and Fe *L* edges. EELS data were acquired with 0.2 s/pixel and 0.1 eV/pixel dispersion. High-resolution STEM images were acquired at 300 kV acceleration voltage using a convergence semi-angle α of 21 mrad, 50 pA probe current and a collection angle of 29-160 mrad for high angle annular dark field (HAADF) imaging, and 0-20 mrad for bright field imaging (BF). Image processing was performed using an open source HyperSpy Python software package [2]. Simulated electron diffraction data as well as high-resolution STEM images were calculated using the JEMS software [3].

S2. Chemico-physical characterization

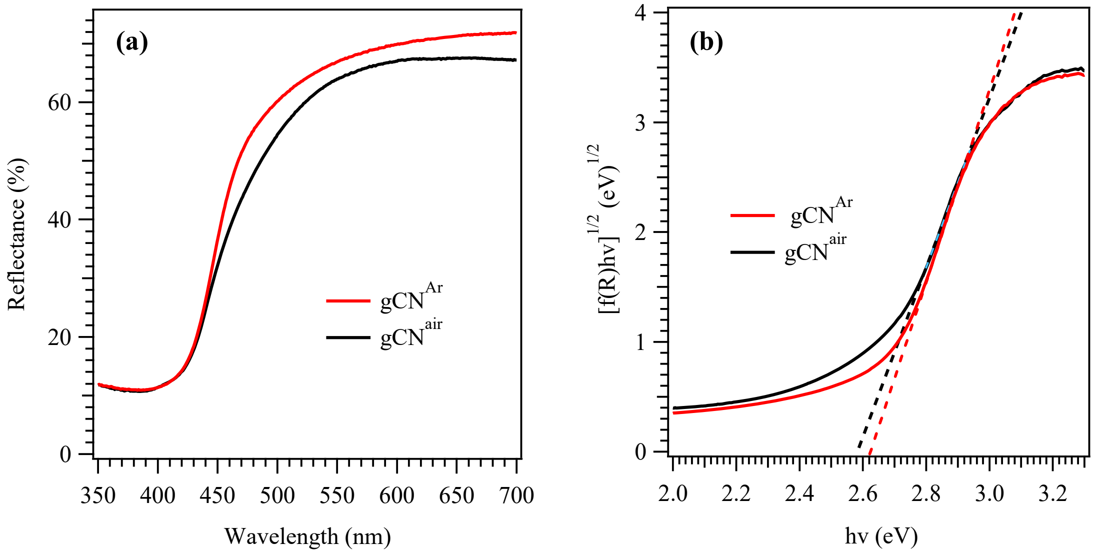

Figure S2. (a) Diffuse reflectance spectrum and (b) corresponding Tauc plot for gCN^{air} powders.

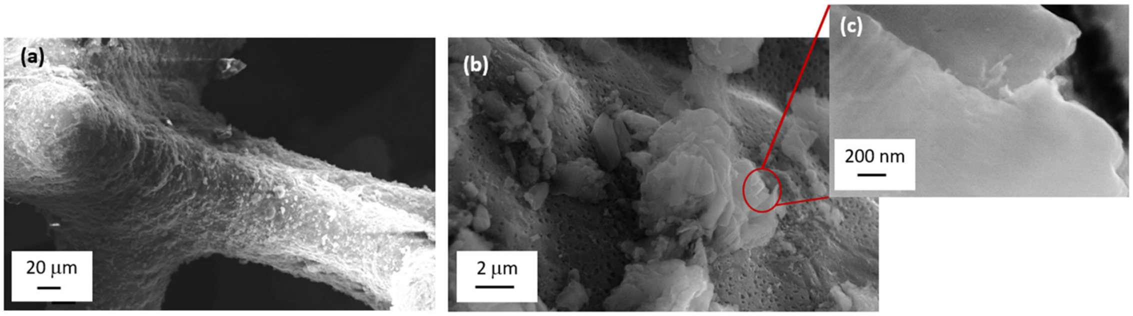

Figure S3. FE-SEM micrographs at different magnification levels for sample gCN^{Ar}.

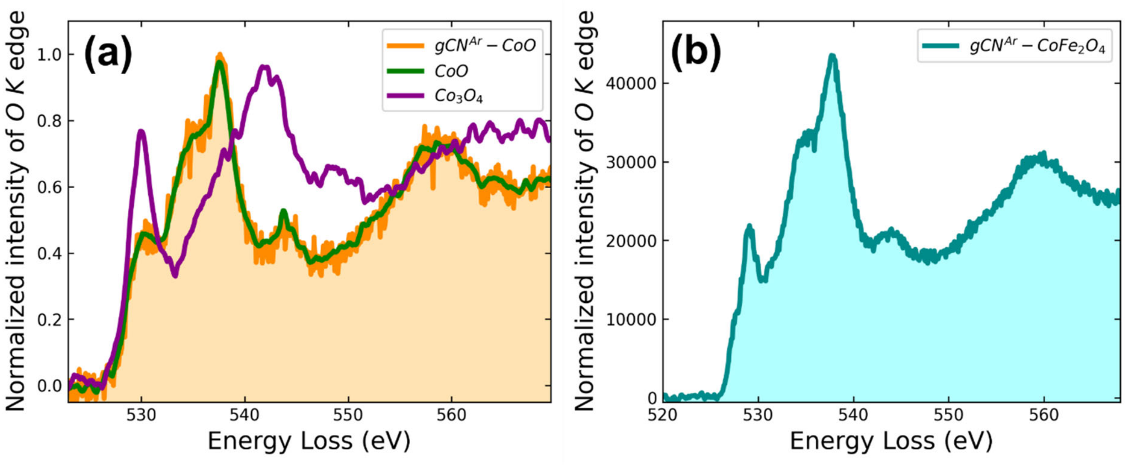

Figure S4. (a) O K edge EELS spectrum acquired on a Co-containing nanoparticle (orange) for gCN^{Ar}-CoO, compared to reference spectra taken on powders of CoO (green) and Co_3O_4 (purple). (b) EELS spectrum of the O K edge collected on specimen gCN-$CoFe_2O_4$.

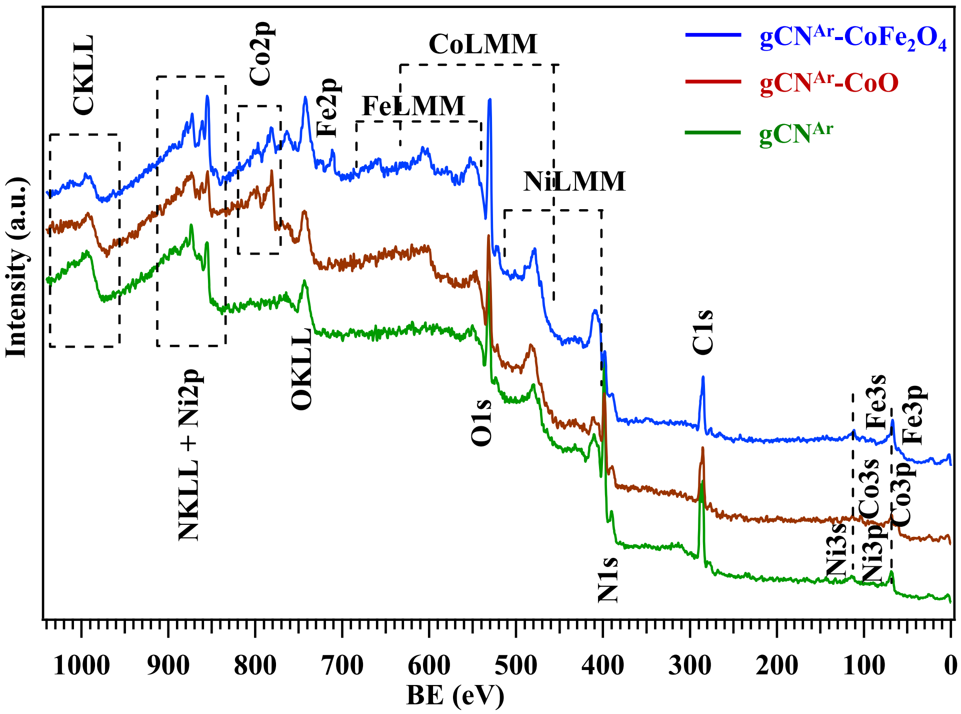

Figure S5. Wide-scan XPS spectra of gCN^{Ar} deposits before and after functionalization with CoO and $CoFe_2O_4$. Quantitative analyses yielded the following atomic percentage ratios: N/C = 1.0, 0.8 and 0.7, for gCN^{Ar}, gCN^{Ar}-CoO and gCN^{Ar}-$CoFe_2O_4$; Co/N = 0.20, for both gCN^{Ar}-CoO and gCN^{Ar}-$CoFe_2O_4$; Fe/N = 0.41, for gCN^{Ar}-$CoFe_2O_4$; Co/Fe = 0.50, for gCN^{Ar}-$CoFe_2O_4$. Co atomic percentage (at.%) values were estimated to be 7.0 and 4.0 % for gCN^{Ar}-CoO and gCN^{Ar}-$CoFe_2O_4$, respectively. Calculation was performed excluding the adventitious carbon component.

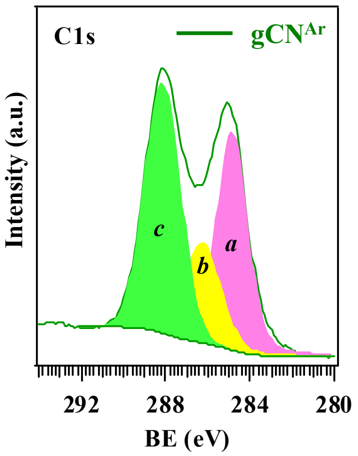

Figure S6. C1s peaks for bare gCN^{Ar} supported on Ni foam.

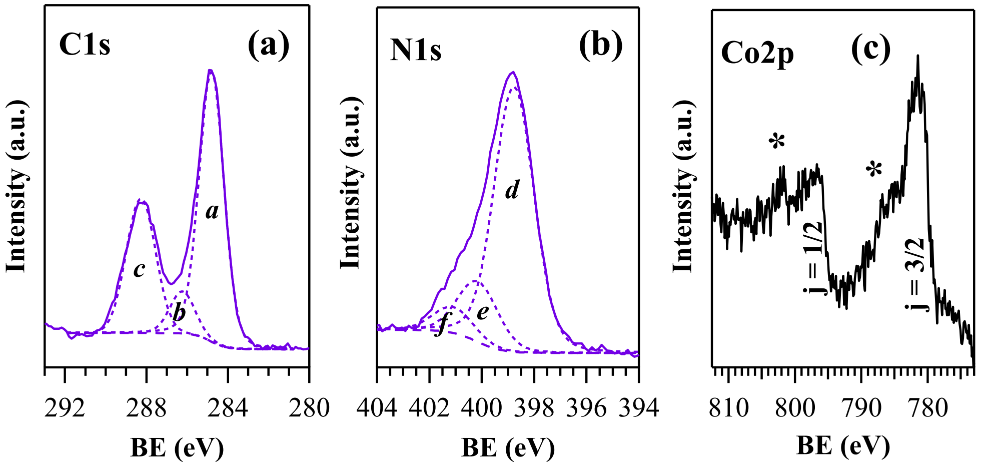

Figure S7. C1s (a) and N1s (b) photopeaks for gCN^{air} along with Co2p (c) signal for gCN^{air}-CoPi. In (c), stars (*) indicate shake-up peaks. Co atomic percentage (at.%) was estimated to be 2.0 %. Calculation was performed excluding the adventitious carbon component.

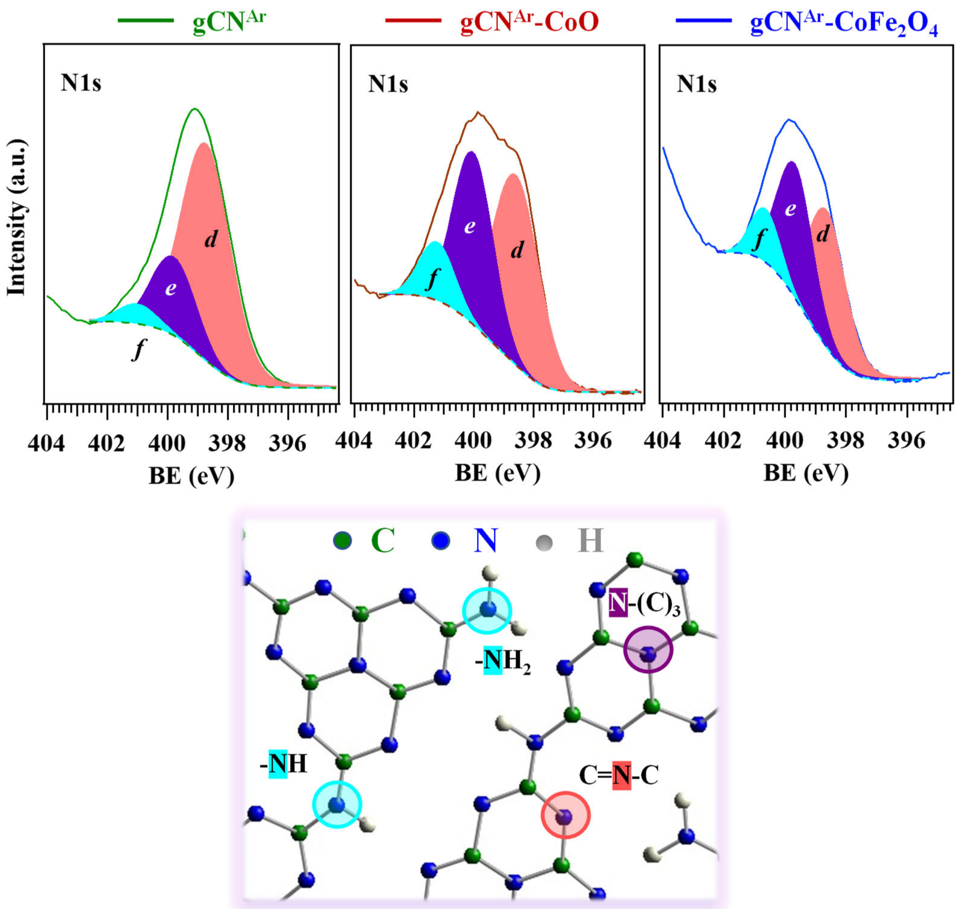

Figure S8. Top panel: N1s photopeaks for gCN^{Ar} deposits on Ni foams before and after functionalization with CoO and $CoFe_2O_4$. Bottom panel: Schematic representation of gCN structure [4], in which non-equivalent N sites are marked. Color codes as in top panel.

The N1s signals resulted from three contributing bands (Figures S7b and S8, top panel): ***d***, the predominant one, due to attributable to N centers in C=N-C moieties (BE = 398.6 eV), [4-12]; ***e***, ascribed to tri-coordinated N atoms in N-$(C)_3$ moieties (BE = 399.8 eV) [8,13-20]; ***f***, due to carbon atoms in uncondensed C-NH_x (x = 1, 2) groups on gCN ring edges (BE = 401.0 eV) [9,10,13-15,17,19] (see Figure S8, bottom panel). The percentage contribution of component ***f*** to the overall N1s signal was evaluated to be 4.9, 11.0, and 12.4 % for gCN^{Ar}, gCN^{Ar}-CoO, and gCN^{Ar}-$CoFe_2O_4$ specimens, respectively.

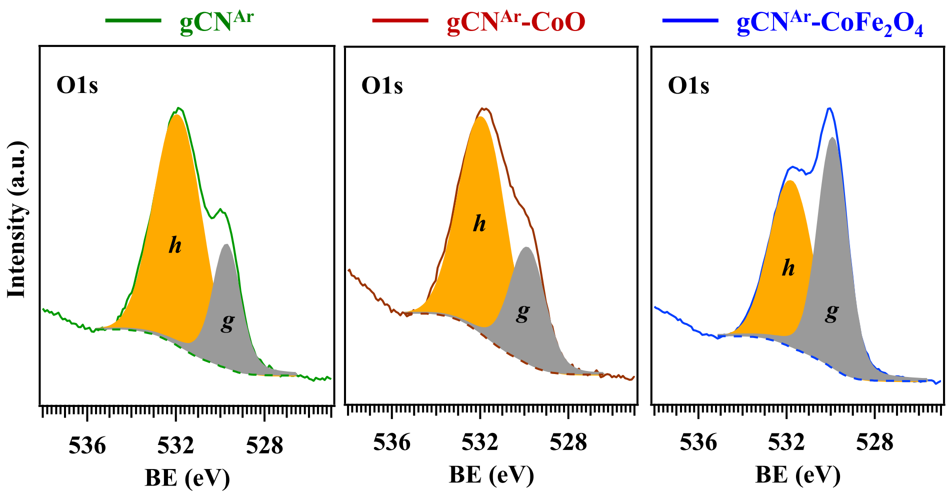

Figure S9. O1s photopeaks for gCN deposits supported on Ni foams before and after functionalization with CoO and $CoFe_2O_4$.

The O1s peak could be fitted by two bands (Figure S9): ***g***, centered at BE = 529.8 eV, related to NiO from the Ni foam substrate [21], as well as to oxygen in CoO and $CoFe_2O_4$ networks, for functionalized specimens [22-26]; ***h***, centered at BE= 531.9 eV, assigned to -OH groups chemisorbed onto N vacancies [5,7,14,16,26,27]. The percentage contribution of bands ***g*** (***h***) to the overall O1s signal were 26.9% (73.1%), for gCN^{Ar}; 30.5% (69.5%), for gCN^{Ar}-CoO; 52.1% (47.9%), for gCN^{Ar}-$CoFe_2O_4$.

S3. Functional tests

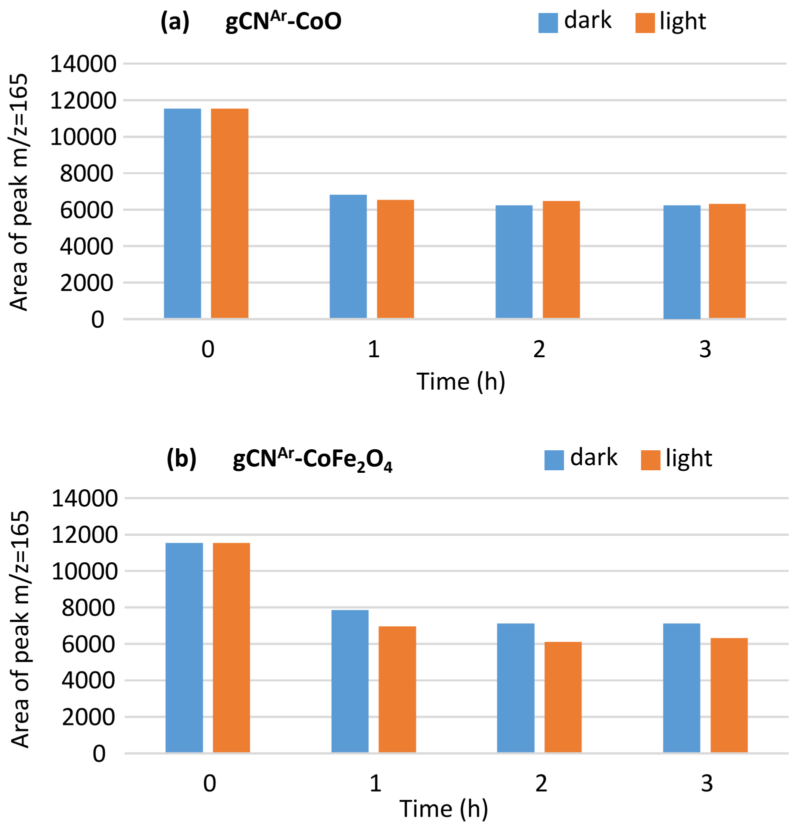

Figure S10. Determination of potassium hydrogen phthalate (KHP) concentration *vs.* time for (a) gCN^{Ar}-CoO and (b) gCN^{Ar}-$CoFe_2O_4$. Quantification of KHP was carried out by measuring the m/z = 165 peak area by flow injection analysis - electrospray mass spectrometry (FIA-ESI/MS). Both samples showed a comparable degradation efficiency in the dark and under illumination, in line with their modest photoactivity (see the main paper text).

References

1. Benedet, M.; Rizzi, G.A.; Gasparotto, A.; Gauquelin, N.; Orekhov, A.; Verbeeck, J.; Maccato, C.; Barreca, D. Functionalization of carbon nitride systems by cobalt and cobalt-iron oxides boosts solar water oxidation performances. *Appl. Surf. Sci.* **2023**, *618*, 156652. https://doi.org/10.1016/j.apsusc.2023.156652.
2. de la Peña, F.; et al. HyperSpy v.1.1.2 (2016). Available online: https://hyperspy.org. https://doi.org/10.5281/zenodo.60697. .
3. Stadelmann, P.A. JEMS—EMS java version, 2004.
4. Niu, P.; Yin, L.-C.; Yang, Y.-Q.; Liu, G.; Cheng, H.-M. Increasing the visible light absorption of graphitic carbon nitride (Melon) photocatalysts by homogeneous self-modification with nitrogen vacancies. *Adv. Mater.* **2014**, *26*, 8046-8052. https://doi.org/10.1002/adma.201404057.
5. Guo, X.; Duan, J.; Wang, W.; Zhang, Z. Modified graphitic carbon nitride as the photocatalyst for wastewater treatment under visible light irradiation. *Fuel* **2020**, *280*, 118544. https://doi.org/10.1016/j.fuel.2020.118544.
6. Dong, F.; Wang, Z.; Li, Y.; Ho, W.-K.; Lee, S.C. Immobilization of polymeric g-C_3N_4 on structured ceramic foam for efficient Visible light photocatalytic air purification with real indoor illumination. *Environ. Sci. Technol.* **2014**, *48*, 10345-10353. https://doi.org/10.1021/es502290f.
7. Zhu, Q.; Qiu, B.; Du, M.; Ji, J.; Nasir, M.; Xing, M.; Zhang, J. Dopant-induced edge and basal plane catalytic sites on ultrathin C_3N_4 nanosheets for photocatalytic water reduction. *ACS Sustainable Chem. Eng.* **2020**, *8*, 7497-7502. https://doi.org/10.1021/acssuschemeng.0c02122.
8. Liang, Q.; Li, Z.; Huang, Z.-H.; Kang, F.; Yang, Q.-H. Holey graphitic carbon nitride nanosheets with carbon vacancies for highly improved photocatalytic hydrogen production. *Adv. Funct. Mater.* **2015**, *25*, 6885-6892. https://doi.org/10.1002/adfm.201503221.
9. Li, Y.; Wang, R.; Li, H.; Wei, X.; Feng, J.; Liu, K.; Dang, Y.; Zhou, A. Efficient and stable photoelectrochemical seawater splitting with TiO_2@g-C_3N_4 nanorod arrays decorated by Co-Pi. *J. Phys. Chem. C* **2015**, *119*, 20283-20292. https://doi.org/10.1021/acs.jpcc.5b05427.
10. Nasri, A.; Jaleh, B.; Nezafat, Z.; Nasrollahzadeh, M.; Azizian, S.; Jang, H.W.; Shokouhimehr, M. Fabrication of g-C_3N_4/Au nanocomposite using laser ablation and its application as an effective catalyst in the reduction of organic pollutants in water. *Ceram. Int.* **2021**, *47*, 3565-3572. https://doi.org/10.1016/j.ceramint.2020.09.204.
11. Morgan, D.J. Core-level reference spectra for bulk graphitic carbon nitride (g-C_3N_4). *Surf. Sci. Spectra* **2021**, *28*, 014007. https://doi.org/10.1116/6.0001083.
12. Kim, C.; Cho, K.M.; Park, K.; Kim, K.H.; Gereige, I.; Jung, H.-T. Ternary hybrid aerogels of g-C_3N_4/α-Fe_2O_3 on a 3D graphene network: an efficient and recyclable Z-scheme photocatalyst. *ChemPlusChem* **2020**, *85*, 169-175. https://doi.org/10.1002/cplu.201900688.
13. Miller, T.S.; Jorge, A.B.; Suter, T.M.; Sella, A.; Corà, F.; McMillan, P.F. Carbon nitrides: synthesis and characterization of a new class of functional materials. *Phys. Chem. Chem. Phys* **2017**, *19*, 15613-15638. https://doi.org/10.1039/C7CP02711G.
14. Fu, J.; Zhu, B.; Jiang, C.; Cheng, B.; You, W.; Yu, J. Hierarchical porous O-doped g-C_3N_4 with enhanced photocatalytic CO_2 reduction activity. *Small* **2017**, *13*, 1603938. https://doi.org/10.1002/smll.201603938.
15. Wang, J.; Zhang, W.-D. Modification of TiO_2 nanorod arrays by graphite-like C_3N_4 with high visible light photoelectrochemical activity. *Electrochim. Acta* **2012**, *71*, 10-16. https://doi.org/10.1016/j.electacta.2012.03.102.
16. Yao, S.-Y.; Chang, K.-S. Solvothermal synthesis of various C_3N_4 films on FTO substrates and their photocatalytic and sensing applications. *J. Am. Ceram. Soc.* **2021**, *104*, 722-732. https://doi.org/10.1111/jace.17484.
17. Peng, G.; Xing, L.; Barrio, J.; Volokh, M.; Shalom, M. A general synthesis of porous carbon nitride films with tunable surface area and photophysical properties. *Angew. Chem. Int. Ed.* **2018**, *57*, 1186-1192. https://doi.org/10.1002/anie.201711669.

18. Wei, F.; Liu, Y.; Zhao, H.; Ren, X.; Liu, J.; Hasan, T.; Chen, L.; Li, Y.; Su, B.-L. Oxygen self-doped g-C_3N_4 with tunable electronic band structure for unprecedentedly enhanced photocatalytic performance. *Nanoscale* **2018**, *10*, 4515-4522. https://doi.org/10.1039/C7NR09660G.
19. Yu, H.; Shi, R.; Zhao, Y.; Bian, T.; Zhao, Y.; Zhou, C.; Waterhouse, G.I.N.; Wu, L.-Z.; Tung, C.-H.; Zhang, T. Alkali-assisted synthesis of nitrogen deficient graphitic carbon nitride with tunable band structures for efficient visible-light-driven hydrogen evolution. *Adv. Mater.* **2017**, *29*, 1605148. https://doi.org/10.1002/adma.201605148.
20. Ren, Y.; Feng, D.; Yan, Z.; Sun, Z.; Zhang, Z.; Xu, D.; Qiao, C.; Chen, Z.; Jia, Y.; Chan Jun, S.; et al. Interfacial coupled engineering of plasmonic amorphous MoO_{3-x} nanodots/g-C_3N_4 nanosheets for photocatalytic water splitting and photothermal conversion. *Chem. Eng. J.* **2023**, *453*, 139875. https://doi.org/10.1016/j.cej.2022.139875.
21. Available online: http://srdata.nist.gov/xps.
22. Chagas, C.A.; de Souza, E.F.; de Carvalho, M.C.N.A.; Martins, R.L.; Schmal, M. Cobalt ferrite nanoparticles for the preferential oxidation of CO. *Appl. Catal., A* **2016**, *519*, 139-145. https://doi.org/10.1016/j.apcata.2016.03.024.
23. Barreca, D.; Gasparotto, A.; Lebedev, O.I.; Maccato, C.; Pozza, A.; Tondello, E.; Turner, S.; Van Tendeloo, G. Controlled vapor-phase synthesis of cobalt oxide nanomaterials with tuned composition and spatial organization. *CrystEngComm* **2010**, *12*, 2185-2197. https://doi.org/10.1039/B926368N.
24. Guo, F.; Shi, W.; Wang, H.; Han, M.; Li, H.; Huang, H.; Liu, Y.; Kang, Z. Facile fabrication of a CoO/g-C_3N_4 p–n heterojunction with enhanced photocatalytic activity and stability for tetracycline degradation under visible light. *Catal. Sci. Technol.* **2017**, *7*, 3325-3331. https://doi.org/10.1039/C7CY00960G.
25. Hu, F.; Luo, W.; Liu, C.; Dai, H.; Xu, X.; Yue, Q.; Xu, L.; Xu, G.; Jian, Y.; Peng, X. Fabrication of graphitic carbon nitride functionalized P–$CoFe_2O_4$ for the removal of tetracycline under visible light: Optimization, degradation pathways and mechanism evaluation. *Chemosphere* **2021**, *274*, 129783. https://doi.org/10.1016/j.chemosphere.2021.129783.
26. Ismael, M.; Wark, M. Photocatalytic activity of $CoFe_2O_4$/g-C_3N_4 nanocomposite toward degradation of different organic pollutants and their inactivity toward hydrogen production: The role of the conduction band position. *FlatChem* **2022**, *32*, 100337. https://doi.org/10.1016/j.flatc.2022.100337.
27. Benedet, M.; Rizzi, G.A.; Gasparotto, A.; Lebedev, O.I.; Girardi, L.; Maccato, C.; Barreca, D. Tailoring oxygen evolution performances of carbon nitride systems fabricated by electrophoresis through Ag and Au plasma functionalization. *Chem. Eng. J.* **2022**, *448*, 137645. https://doi.org/10.1016/j.cej.2022.137645.
